# Neuronal synaptic autoantibodies and PsycHosis: Prognostic factors for the diagnosis of autoimmune encephalitis in patients with psychotic spectrum disorders -The PHLAMES Study

**DOI:** 10.1192/j.eurpsy.2025.236

**Published:** 2025-08-26

**Authors:** A. Pigoni

**Affiliations:** Neuroscience, IRCCS Fondazione Ca Granda Policlinico Hospital, Milan, Italy

## Abstract

**Abstract: Background:**

The recent years witnessed an increase in the knowledge regarding autoimmune encephalitis (AE). These autoimmune entities often present with mixed psychiatric and neurologic features and in up to 4% of the cases the presentation is purely psychiatric. The diagnosis can be made only through the discovery of Neuronal Surface Autoantibodies (NSAbs) in the Cerebro Spinal Fluid, but symptoms and signs of possible and probable diagnosis have been described (Pollack et al., 2020). However, NSAbs can be found also in peripheral blood in various percentage of patients. The role of these antibodies in psychiatric patients is yet not known.

The PHLAMES study aims at evaluating first episode psychosis (FEP) patients for signs and symptoms of AE in a psychiatric setting, with the double objective of assessing the diagnosis of AE and the role of circulating NSAbs in psychiatric patients, through clinical evaluation, biological samples, and neuroimaging.

**Methods:**

In the PHLAMES study, all patients with FEP (<6 months from the onset) were tested with a diagnostic algorithm for signs or symptoms of AE. A complete psychiatric and neurologic assessment was performed; cognitive tests were administered. All patients underwent blood sample to test for circulating auto-antibodies against SNC structures.

A subsample also underwent MRI, including gadolinium contrast.

Analyses compared patients testing positive for serum NSAbs (NSAbs-POS) to those tested negative (NSAbs-Neg).

**Results:**

12.8% of the patients tested positive for serum NSAbs (NSAbs-POS). No difference in terms of age, sex, BMI, years od education, and ethnicity was found between groups.

Regarding the neurologic variables, NSAbs-POS significantly showed more memory deficits, parkinsonism signs, and speech disorders (p<0.001), compared to NSAbs-NEG patients. Similarly, NSAbs-POS patients presented a significant in TMT-A, Raven, and RAVLT scores (p<0.05) compared to NSAbs-NEG. Finally, NSAbs-POS patients presented an increased score at PANSS **“**Somatic implication**”** item and a reduced score at PANSS **“**Insight**”** (p<0.05) items, suggesting a higher concern of these patients regarding their help and a greater awareness of their condition.

Regarding MRI, differences are present between groups, both on structural and on contrasted images.

**Discussion:**

Our preliminary findings suggest the possibility that NSAbs-POS patients might represent a subpopulation of FEP with specific characteristics. These results are preliminary and need a confirmation in bigger samples, but they might represent a step towards the identification of clinically meaningful subgroups in FEP defined through an easy and not invasive test, helping to dissect the heterogeneity of psychiatric disorders and moving towards precision psychiatry.

**Disclosure of Interest:**

None Declared

